# Macrolide-Resistant *Mycoplasma pneumoniae* Infections in Children, Ohio, USA

**DOI:** 10.3201/eid2706.203206

**Published:** 2021-06

**Authors:** Mariana M. Lanata, Huanyu Wang, Kathy Everhart, Melisa Moore-Clingenpeel, Octavio Ramilo, Amy Leber

**Affiliations:** Nationwide Children’s Hospital, Columbus, Ohio, USA

**Keywords:** *Mycoplasma pneumoniae*, macrolide sensitivity, macrolide resistance, molecular detection, children, disease severity, community-acquired pneumonia, bacteria, Ohio, United States, antimicrobial resistance, respiratory infections, pneumonia

## Abstract

Emergence of macrolide-resistant *Mycoplasma pneumoniae* (MRMp) challenges empiric macrolide therapy. Our goal was to determine MRMp rates and define characteristics of children infected with macrolide-sensitive *M. pneumoniae* (MSMp) versus MRMp in Ohio, USA. We cultured PCR-positive *M. pneumoniae* specimens and sequenced *M. pneumoniae*–positive cultures to detect macrolide resistance mutations. We reviewed medical records to compare characteristics of both groups. We identified 14 (2.8%) MRMp and 485 (97.2%) MSMp samples. Patients in these groups had similar demographics and clinical characteristics, but patients with MRMp had longer hospitalizations, were more likely to have received previous macrolides, and were more likely to have switched to alternative antimicrobial drugs. MRMp-infected patients also had ≈5-fold greater odds of pediatric intensive care unit admission. Rates of MRMp infections in children in central Ohio are low, but clinicians should remain aware of the risk for severe illness caused by these pathogens.

*Mycoplasma pneumoniae* is a major pathogen that accounts for up to 40% of the total number of community-acquired pneumonias (CAPs) in children and up to 19% of the pediatric CAPs that require hospitalization (*1*,*2*), yet those numbers might not reflect its actual clinical impact because testing for *M. pneumoniae* is not performed routinely. *M. pneumoniae* infection has a wide range of manifestations, from asymptomatic infection to severe pneumonia requiring admission to the intensive care unit (*3*,*4*). Because it lacks a cell wall, *M. pneumoniae* is not susceptible to β-lactam antimicrobial drugs, which are first-line therapy for CAP in children (*5*).

Macrolides are considered the antimicrobial drugs of choice for the treatment of *M. pneumoniae* infections in children (*5*); however, in the past few decades, macrolide-resistant *M. pneumoniae* (MRMp) has emerged. Rates of resistance are highest in Asia, as high as 100%, and reported rates in the United States vary from 3.5% to 13.2% (*3*,*6*–*13*). No published data are available from Ohio, where we conducted our study.

Macrolide resistance is conveyed by single base mutations in the V region of 23S rRNA, which codes for the binding site of macrolides in the *M. pneumoniae* ribosome. The most common mutations include the change of A to C/G/T at location A2063 or at location A2064 (*14*,*15*). These are the 2 mutations associated with macrolide resistance that have been reported in the United States (*3*,*6*).

*M. pneumoniae* is a slow-growing, fastidious organism, making routine culture and phenotypic antimicrobial drug sensitivity testing impractical for clinical use and limiting the use of these techniques mainly to research purposes. Since molecular assays were developed, diagnosis of *M. pneumoniae* infections has shifted from serology to molecular detection using PCR, resulting in improved sensitivity and specificity. However, even with molecular detection, most clinicians have no information regarding antimicrobial sensitivity of *M. pneumoniae*. Therefore, as in many other settings, we currently have no data on local rates of MRMp, and most children diagnosed with *M. pneumoniae* infection are treated initially with macrolides. If clinical concerns for macrolide resistance occur while children are receiving therapy, clinicians sometimes choose to switch antimicrobial therapy to another agent, although there are no established clinical parameters or guidelines concerning when to consider potential resistance to macrolide antimicrobial drugs.

Studies, emerging mainly from Asia, have reported increased disease severity in adults and children infected with MRMp. More consistently, studies have demonstrated longer duration of fever in patients infected with MRMp and longer duration of hospitalization. Other studies have reported more frequent pulmonary complications and the need for changing antimicrobial drug therapy (*16*–*19*). Chen et al. recently published a meta-analysis further consolidating the evidence for longer duration of fever and hospitalization in patients with MRMp, but no differences were reported in clinical presentation, laboratory results, or chest radiograph findings (*20*). Data about MRMp infection in children in the United States remain limited.

The primary objective of this study was to determine the rate of MRMp infections in children in central Ohio, USA. Our second objective was to examine the clinical characteristics, antimicrobial drug treatment, and outcomes in this cohort; to identify potential differences between patients infected with macrolide-sensitive *M. pneumoniae* (MSMp) and those infected with MRMp; and to determine whether infection with MRMp was associated with worse clinical outcomes than infection with MSMp.

## Methods

### Study Samples

We collected a retrospective convenience sample from standard-of-care clinical samples with orders for *M. pneumoniae* molecular testing performed either using an in-house laboratory-developed PCR for *M. pneumoniae* (*21*) or the PCR for *M. pneumoniae* included as part of a multiplex PCR panel for respiratory pathogens (BioFire FilmArray Respiratory Panel version 1.7; BioFire, https://www.biofiredx.com). We identified samples positive for *M. pneumoniae* and having adequate remnant volume for further analysis using the laboratory database for October 2015–January 2019. The samples consisted of nasopharyngeal or throat swab specimens collected in M4 transport media.

### Mycoplasma Culture

We stored samples at 4°C pending PCR results. We cultured the samples that tested positive for *M. pneumoniae* within 48 hours of collection in SP4 glucose broth (Remel, http://www.remel.com) and incubated them at 35°C until isolates grew, or for a maximum of 4 weeks for a negative culture, according to standard procedures (*22*). Culture positivity was identified by color change of the broth and later confirmed by our laboratory-developed *M. pneumoniae* PCR in a subset of patients. We discarded samples displaying bacterial contamination (detected by cloudy or yellow color change).

### Sequencing for Macrolide Resistance Detection

We amplified domain V of the 23S-rRNA (nt 1937–2154; reference strain GenBank accession no. X68422) (*23*) from all positive *M. pneumoniae* cultures, where both point mutations that convey macrolide resistance described in the United States are located. We performed Sanger sequencing on the PCR products and compared the sequences with the corresponding region of the wild-type reference strain (ATCC 15322). We sent 2 de-identified samples to a reference laboratory (Mycoplasma Laboratory, University of Alabama at Birmingham, Birmingham, AL, USA) for phenotypic sensitivity testing to assess the effect of a novel mutation (A2065Δ).

### Clinical and Treatment Characteristics

We reviewed electronic medical records from all patients with sequenced samples. We collected demographic characteristics, including age, gender, race, patient location, any previous medical encounter during illness, and vaccination status, for each patient. We also collected clinical data such as symptoms at the first medical encounter, including fever, cough, rhinorrhea, rash, and central nervous system manifestations. We recorded all diagnostic testing associated with the medical encounter, including chest radiographs and laboratory testing (blood cultures, complete blood counts, other viral testing) and medical interventions, mainly with regard to antimicrobial drug treatment. This study was approved by the Nationwide Children’s Hospital Institutional Review Board (IRB17-01280).

### Statistical Analysis

We assessed group comparisons using χ^2^ or Fisher exact tests for categorical variables and 2-sample *t*-tests with Satterthwaite corrections for unequal group variance where needed or Wilcoxon rank sum tests for continuous variables. We used multivariable logistic regression to evaluate risk factors for binary outcomes (hospital admission, pediatric intensive care unit [PICU] admission, presence of fever, hypoxemia), with a Firth correction for small sample size when warranted. We used negative binomial regression to evaluate risk factors for continuous outcomes (duration of hospitalization, duration of fever); results have been exponentiated to reflect risk ratios. For all multivariable models, when sample size allowed, we ran separate models for the full cohort as well as for the cohort with viral testing, because of the more limited number of patients who received viral testing; results were similar for both cohorts. We ran separate models for collinear covariates and presented the model with the best goodness of fit (based on the Akaike Information Criterion). We based variable selection for all multivariable models on backward stepwise selection, with an entry criterion of p<0.15; we retained resistance in all models regardless of statistical significance because it was the primary risk factor. We used SAS 9.4 (https://www.sas.com) to conduct all analyses. 

## Results

### Detection of Macrolide Resistance Mutations by Sequencing

During October 2015–January 2019, a total of 744 samples identified as *M. pneumoniae*–positive by PCR were cultured for isolation of *M. pneumoniae*. Among these, 553 (74.3%) yielded a positive *M. pneumoniae* culture (Figure).

**Figure:**
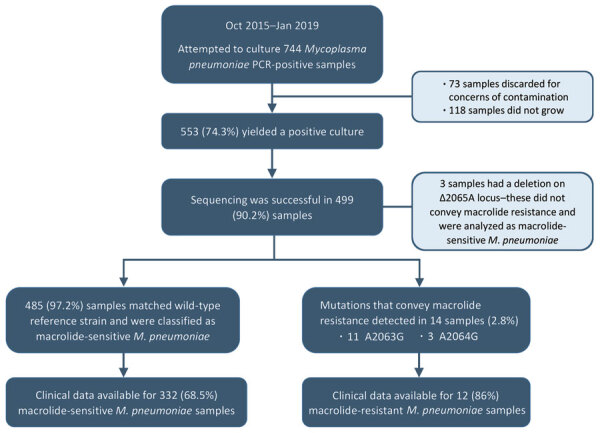
Flowchart for selection of *Mycoplasma pneumoniae*-positive respiratory samples for macrolide resistance testing and children with available information for analysis of clinical variables in study of children infected with *M. pneumoniae*, Ohio, USA, 2015–2019.

We performed sequencing on those 553 culture-positive samples and were successful with 499 (90.2%). The sequences of the V domain of the 23rRNA from a total of 485 (97.2%) samples matched that of the wild-type reference strain. We detected mutations associated with macrolide resistance in 14 samples (2.8%); of those, 11 corresponded to the A2063G and 3 to A2064G mutations (Figure). We also identified 3 samples with a deletion on A2065, a locus adjacent to the mutations described to convey macrolide resistance.

### Phenotypic Susceptibility Testing

From the 3 samples with the A2065Δ mutation, we were able to regrow only 2 from frozen aliquots and sent them for phenotypic sensitivity testing. Erythromycin, tetracycline, and levofloxacin were the antimicrobial drugs tested. The *M. pneumoniae* isolates from both samples were sensitive to all 3 drugs according to Clinical and Laboratory Standards Institute guidelines; therefore, this deletion was not found to convey resistance to macrolides (*24*). For purpose of the rest of the analysis, we included these samples in the MSMp group.

### Demographic, Clinical, and Treatment Data

Clinical data were available for 344 (68.9%) of the sequenced samples, including 12/14 (86%) of the MRMp isolates and 332/485 (68.5%) of the MSMp isolates. Both groups of patients had similar demographics, including the presence of concurrent conditions, previous medical encounters, and vaccination status (Table 1). Of the concurrent conditions, asthma was present in 50% of patients. No differences were found in the clinical characteristics at the time of presentation to medical care between both groups, with the exception of maximum temperature among patients who had fever; those in the MRMp group had a lower maximum temperature. Central nervous system manifestations appeared to be more common in the MRMp group; however, this finding was not statistically significant.

**Table 1:** Patient demographics and clinical characteristics of children infected with macrolide-sensitive and macrolide-resistant strains of *Mycoplasma pneumoniae*, Ohio, USA, 2015–2019*

Characteristic	MRMp, n = 12	MSMp, n = 332	p value
Sex			0.9046
M	7 (55)	175 (53)	
F	5 (45)	157 (47)	
Year			0.6508
2015	4 (33)	146 (44)	
2016	1 (8)	51 (15)	
2017	2 (17)	52 (16)	
2018	5 (42)	73 (22)	
2019	0	10 (3)	
Age, y, mean (SD)	9.13 (3.74)	8.73 (4.62)	0.7683
Previous visits			
Any previous visits	8 (67)	188 (57)	0.4901
No. previous visits, median (IQR)	1.5 (1–2)	1 (1–2)	0.3491
Underlying conditions	6 (50)	139 (42)	0.5752
Chronic immunosuppression	1 (8)	5 (2)	0.1392
Up to date in vaccines	12 (100)	316 (97)	>0.9999
Inpatient	6 (50)	130 (39)	0.551
Median duration of hospitalization, d (IQR)	8 (6–10)	2 (1.5–3)	**0.0132**
PICU	3 (25)	21 (6)	**0.0433**
Median duration of PICU stay, d (IQR)	2 (1–8)	2 (1.5–5)	0.8596
Hypoxemia	3 (25)	73 (23)	>0.9999
Median duration of O_2_ support, d (IQR)	4 (1–8.5)	1 (0.5–2)	0.1302
Mechanical ventilation	1 (33)	6 (8)	0.2487
Median duration of symptoms, d (IQR)	7 (6.5–13)	7 (5–10)	0.3324
Fever	11 (92)	271 (81)	0.7024
Median duration of fever, d (IQR)	4 (2–7)	4 (2–7)	0.984
Median maximum temperature, °C (IQR)	38.4 (38.1–39.4)	39.1 (38.4–39.7)	**0.0415**
Fatigue	5 (42)	91 (27)	0.3298
Decreased appetite/oral food intake	4 (33)	125 (38)	>0.9999
Decreased urine output	0	22 (7)	>0.9999
Cough	12 (100)	320 (96)	>0.9999
Shortness of breath/respiratory distress	5 (42)	91 (27)	0.3268
Sore throat	0	67 (20)	0.1332
CNS manifestation	2 (17)	10 (3)	0.0607
Nausea/vomiting	1 (8)	55 (17)	0.6987
Diarrhea	1 (8)	30 (9)	>0.9999
Rash	0	25 (7.5)	>0.9999

*Values are no. (%) except as indicated. Bold type indicates statistical significance. CNS, central nervous system; IQR, interquartile range; MRMp, macrolide-resistant *Mycoplasma pneumoniae*; MSMp, macrolide-sensitive *Mycoplasma pneumoniae*; PICU, pediatric intensive care unit.

Among patients for whom chest radiographs were obtained (12 in the MRMp group and 264 in the MSMp group), most of their radiographs were abnormal (100% of MRMp and 95% of MSMp) (Table 2). Of the 344 patients with clinical data, 91 received their diagnoses by multiplex PCR panel alone, 168 by in-house *M. pneumoniae* PCR alone, and 85 with both. Therefore, viral testing was available in the subset of 176 patients in the cohort tested using the multiplex panel: 10 (83%) patients with MRMp and 166 (50%) patients with MSMp. Viruses identified included rhinovirus/enterovirus, adenovirus, respiratory syncytial virus, coronavirus, influenza B, and parainfluenza types 2 and 3. Although co-detection of viruses was more common in the MSMp group, this difference was not statistically significant. No bacterial co-infections were detected (Table 2).

**Table 2:** Respiratory examination, chest radiograph findings, and results of viral testing in children infected with *Mycoplasma pneumoniae*, Ohio, USA, 2015–2019*

Test	MRMp, n = 12	MSMp, n = 332	p value
Respiratory examination			
Abnormal	9 (75)	214 (64)	0.5513
Crackles	3 (25)	96 (45)	0.7344
Rales	1 (11)	25 (12)	>0.9999
Rhonchi	1 (11)	23 (11)	>0.999
Decreased breath sounds	5 (56)	104 (49)	0.7441
Wheezing	1 (11)	51 (24)	0.6888
Chest radiograph findings	n = 12	n = 261	>0.9999
Abnormal	12 (100)	243 (93.1)	
Focal consolidation	3 (25)	170 (65.1)	
Multifocal consolidation	4 (33)	41 (15.7)	
Peribronchial thickening	2 (17)	45 (17.2)	
Atelectasis	0	8 (3.1)	
Pleural effusion	1 (11)	39 (14.9)	
Viral testing done by multiplex PCR	10 (83.3)	166 (50)	0.4086
*M. pneumoniae* alone	9 (90)	118 (71)	
*M. pneumoniae* + 1 virus	1 (10)	37 (22)	
*M. pneumoniae* + >2 viruses	0	11 (7)	

*Values are no. (%) except as indicated. MRMp, macrolide-resistant *Mycoplasma pneumoniae*; MSMp, macrolide-sensitive *Mycoplasma pneumoniae*

Macrolide resistance was not significantly associated with duration of fever in univariate (relative risk [RR] 0.99, 95% CI 0.66–1.46) or multivariable (adjusted RR [aRR] 0.93, 95% CI 0.66–1.30) analysis. However, older age (aRR 1.02 [95% CI 1.0–1.03]) per 1 year increase in age), previous medical visit (aRR = 1.71, 95% CI 1.44–2.04), and previous antimicrobial drug treatment (aRR 1.21, 95% CI 1.03–1.42) were significantly associated with longer duration of fever.

When we evaluated outcomes related to disease severity, MRMp infection was not a risk factor for hospitalization. In multivariable analyses, younger age, presence of concurrent conditions, previous medical encounters, presence of abnormal respiratory examination, and preceding therapy with a nonmacrolide antimicrobial drug were significantly associated with increased odds for hospitalization (Table 3). Among the subset of patients who had viral testing performed, positive viral co-detection was associated with significantly lower odds of hospitalization. We also examined for variables associated with duration of hospitalization and found that, in univariate analysis, patients infected with MRMp strains had significantly longer duration of hospitalization than those infected with MSMp (Table 4); no other study variables were significantly associated with duration of hospitalization.

**Table 3:** Risk factors for hospital admission in children infected with *Mycoplasma pneumoniae*, Ohio, USA, 2015–2019*

Risk factor	Univariate analysis			Multivariable analysis		
	OR (95% CI)	p value		aOR (95% CI)		p value
Female sex	0.99 (0.64–1.53)	0.9599				
Age, y	0.96 (0.92–1.01)	0.1125		0.928 (0.877–0.982)		0.0096
Underlying condition	3.47 (2.21–5.46)	**<0.0001**		4.234 (2.506–7.155)		<0.0001
Any previous visit	3.6 (2.24–5.78)	**<0.0001**		2.094 (1.131–3.877)		0.0188
Macrolide resistance	1.55 (0.49–4.92)	0.4547		1.171 (0.324–4.231)		0.8094
Abnormal respiratory examination	4.31 (2.55–7.26)	**<0.0001**		4.063 (2.291–7.204)		<0.0001
Previous treatment with antimicrobial drugs	3.03 (1.9–4.83)	**<0.0001**		2.606 (1.377–4.934)		0.0033
Previous nonmacrolide antimicrobial drugs	2.85 (1.78–4.56)	**<0.0001**				
Positive viral test	0.24 (0.08–0.68)	**0.0071**				
MRMp and positive viral test	0.23 (0.04–1.44)	0.1166				

*Bold type indicates statistical significance. aOR, adjusted OR; MRMp: macrolide-resistant *Mycoplasma pneumoniae*; OR, odds ratio.

**Table 4:** Risk factors for longer duration of hospitalization in children hospitalized with *Mycoplasma pneumoniae* infection, Ohio, USA, 2015–2019*

Risk factor	Univariate analysis			Multivariable analysis	
	RR (95% CI)	p value		aRR (95% CI)	p value
Female sex	0.85 (0.63–1.15)	0.2852			
Age, y	0.98 (0.95–1.01)	0.2213			
Underlying condition	1.25 (0.92–1.71)	0.1592			
Any previous visit	0.83 (0.58–1.16)	0.2734			
Macrolide resistance	2.91 (1.56–5.44)	**0.0008**		2.04 (0.97–4.32)	0.061
Abnormal respiratory examination	1.14 (0.77–1.7)	0.5103			
Previous treatment with antimicrobial drugs	0.94 (0.69–1.27)	0.6882			
Previous nonmacrolide antimicrobial drugs	0.97 (0.71–1.31)	0.8204			
Positive viral test	0.96 (0.69–1.34)	0.8122			
MRMp and positive viral test	1.67 (0.51–5.44)	0.3946			

*Bold type indicates statistical significance. aRR, adjusted RR; MRMp, macrolide-resistant *Mycoplasma pneumoniae*; RR, relative risk.

A total of 24 (7% of the cohort, 17.6% of hospitalizations) patients required PICU admission, the majority (n = 21, 87.5%) because of escalated respiratory support; 5 required invasive ventilation, 1 a Venturi mask, and 13 bilevel positive airway pressure. Two patients were admitted because of concerns of severe sepsis, and 1 because of altered mental status. Macrolide resistance was significantly associated with PICU admission (univariate odds ratio [OR] 5.34, 95% CI 1.39–20.55), as was the presence of concurrent conditions, any previous medical visits, abnormal respiratory exam, and previous therapy with a nonmacrolide antimicrobial drug (Table 5). Although too few patients were admitted to the PICU to enable us to perform a comprehensive multivariable analysis, we found that macrolide resistance remained significantly associated with odds of PICU admission after adjusting one at a time for the presence of concurrent conditions, any previous medical visits, abnormal respiratory examination, and previous therapy with a nonmacrolide antimicrobial drug (adjusted OR [aOR] for macrolide resistance 4.9–5.1; Table 6). Presence of macrolide resistance was not associated with increased risk for hypoxemia when adjusted for other factors. Only the presence of concurrent conditions (aOR 3.45; p<0.0001) and any previous medical visits (aOR 2.35; p = 0.0151) were significant risks for hypoxemia and oxygen requirement (Table 7).

**Table 5:** Risk factors for pediatric intensive care unit admission in children infected with *Mycoplasma pneumoniae*, Ohio, USA, 2015–2019*

Risk factor	Univariate analysis	
	OR (95% CI)	p value
Female sex	1.13 (0.5–2.55)	0.7754
Age, y	0.98 (0.9–1.08)	0.7206
Underlying condition	4.32 (1.72–10.88)	**0.0019**
Any previous visit	3.73 (1.31–10.62)	**0.0138**
Macrolide resistance	5.34 (1.39–20.55)	**0.015**
Abnormal respiratory examination	5.34 (1.41–20.21)	**0.0137**
Previous treatment with antimicrobial drugs	3.17 (1.38–7.27)	**0.0066**
Previous nonmacrolide antimicrobial drugs	2.81 (1.23–6.42)	**0.014**
Positive viral test	0.79 (0.31–2.01)	0.6127
MRMp and positive viral test	2.11 (0.27–16.71)	0.4805


*Bold type indicates statistical significance. MRMp, macrolide-resistant *Mycoplasma pneumoniae*; OR, odds ratio.

**Table 6:** Multivariable models for assessing adjusted risk factors for pediatric intensive care unit admission in children infected with *Mycoplasma pneumoniae*, Ohio, USA, 2015–2019*

Model	aOR (95% CI)	p value
Model 1		
Macrolide resistance	5.111 (1.248–20.926)	**0.0233**
Underlying conditions	4.238 (1.68–10.694)	**0.002**
Model 2		
Macrolide resistance	4.964 (1.247–19.762)	**0.023**
Any previous visits	3.625 (1.273–10.323)	**0.0159**
Model 3		
Macrolide resistance	4.895 (1.227–19.532)	**0.0245**
Abnormal respiratory exam	5.164 (1.37–19.461)	**0.0153**
Model 4		
Macrolide resistance	4.969 (1.241–19.906)	**0.0235**
Any previous antimicrobial drugs	3.076 (1.332–7.101)	**0.0085**
Model 5		
Macrolide resistance	4.911 (1.237–19.499)	**0.0237**
Any previous nonmacrolide antimicrobial drugs	2.717 (1.184–6.234)	**0.0183**

*Bold type indicates statistical significance. aOR, adjusted odds ratio.

**Table 7:** Risk factors for hypoxemia in children infected with *Mycoplasma pneumoniae*, Ohio, USA, 2015–2019*

Characteristic	Univariate analysis			Multivariable analysis	
	OR (95% CI)	p value		aOR (95% CI)	p value
Female sex	1.117 (0.68–1.85)	0.6645			
Age, y	0.964 (0.91–1.02)	0.194			
Underlying conditions	3.279 (1.94–5.54)	**<0.0001**		3.46 (2–5.98)	**<0.0001**
Any previous visit	3.202 (1.8–5.69)	**<0.0001**		1.77 (0.94–3.33)	0.0796
Macrolide resistance	1.236 (0.34–4.54)	0.75		0.91 (0.22–3.72)	0.8965
Abnormal respiratory examination	25.288 (6.98–91.64)	**<0.0001**			
Previous treatment with antimicrobial drugs	2.43 (1.45–4.07)	**0.0007**		2.35 (1.18–4.68)	**0.0151**
Previous nonmacrolide antimicrobial drugs	2.129 (1.27–3.58)	**0.0043**			
Positive viral test	0.902 (0.46–1.79)	0.7683			
MRMp and viral positive	0.957 (0.16–5.81)	0.9618			

*Bold type indicates statistical significance. aOR, adjusted odds ratio; MRMp: macrolide-resistant *Mycoplasma pneumoniae*; OR, odds ratio.

### Antimicrobial Drug Treatment

The number of patients with previous antimicrobial drug prescriptions was similar in both groups (41.7% MRMp, 31.9% MSMp; p = 0.53), with a median duration of 3 days (interquartile range [IQR] 2.5–5.7) for MRMp and 4 days (IQR 2–6) for MSMp (p>0.99). Most (96%) of these were antimicrobial drugs not expected to treat *M. pneumoniae* infection. Median time of prescription was on day 5 of illness (IQR 3–9). Three (25%) patients with MRMp versus 7 (2.1%) with MSMp received therapy with azithromycin before the medical encounter (p = 0.0017). Despite the clinician’s lack of knowledge about presence of macrolide resistance, a larger proportion of patients with MRMp infection (25%) than patients with MSMp infection (4.5%) were treated with levofloxacin as the definitive therapy instead of a macrolide (p = 0.0267) (Table 8).

**Table 8:** Antimicrobial drug treatment for children infected with *Mycoplasma pneumoniae*, Ohio, USA, 2015–2019*

Characteristic	MRMp, n = 12	MSMp, n = 332	p value
Patients with previous antimicrobial drug treatment, no. (%)	5 (42)	106 (32)	0.5339
Nonmacrolide	5 (42)	102 (31)	0.5262
Macrolide	3 (25)	7 (2.1)	**0.0017**
Definitive treatment during medical encounter, no. (%)			
Azithromycin	9 (75)	317 (95.5)	**0.0197**
Levofloxacin	3 (25)	15 (4.5)	**0.0267**

*Bold type indicates statistical significance. MRMp, macrolide-resistant *Mycoplasma pneumoniae*; MSMp, macrolide-sensitive *Mycoplasma pneumoniae*.

## Discussion

Macrolide-resistant *M. pneumoniae* infections are becoming more relevant as pathogen-specific testing is increasing and reports of resistance to macrolides are becoming more widespread throughout the world. Resistance rates vary, but the highest reported resistance rates are from countries in Asia, as high as 100% (*3*,*12*). Lower rates have been reported in Europe and South America; few data are available from the United States (*3*,*13*). In today’s globalized world, spread of resistant organisms is common and thus a major concern. Unlike for other bacteria, no cumulative data such as antibiograms are routinely available to help guide empiric therapy for *M. pneumoniae*. Because of *Mycoplasma*’s unique slow growth characteristics and lack of availability of phenotypic susceptibility testing, antimicrobial drug treatment is routinely initiated without any knowledge of macrolide resistance rates.

The ready availability of specific molecular testing for *M. pneumoniae* at Nationwide Children’s Hospital enable us to generate a large convenience sample of patients infected with *M. pneumoniae* for this study. Our data demonstrated a low rate of MRMp of 2.8%. Although this rate of resistance is lower than those previously described in other studies (*6*–*10*), the difference is likely related to differences in study design. In our study, because of the widespread and routine use of testing for *M. pneumoniae* infections in our institution and affiliated urgent care centers, we were able to include a larger sample size of nonselected patients. The resistance mutations detected in our population are similar to others reported worldwide and confirmed that the A2063G mutation was the most commonly found (*8*,*10*,*16*,*25*,*26*). Although it is a novel mutation, a deletion on A2065Δ was detected in 3 isolates, but it did not confer phenotypic resistance to macrolides.

Although children infected with *M. pneumoniae* can have a mild course of disease, some develop severe disease, requiring hospitalization (*1*,*3*,*7*). The presence of mutations associated with macrolide resistance did not affect the need for hospitalization in our study. In the study cohort, 50% of patients infected with MRMp were managed as outpatients. Our findings confirm those of others in different countries (*7*,*18*,*27*,*28*) and a recent surveillance study in the United States (*10*), providing further evidence that children infected with MRMp and MSMp in the United States demonstrated no significant differences in clinical presentation. This finding was also supported in the recent meta-analysis by Chen et al. (*20*). Taken together, these data point to the challenges that clinicians face when treating patients infected with *M. pneumoniae*. Because there is no practical way to identify patients infected with MRMp on the basis of clinical findings, most, if not all, are empirically treated with macrolides and some, albeit a small number, receive ineffective treatment.

Our data indicate that among hospitalized patients infected with MRMp, infections may remain unidentified for days and their conditions may worsen while they receive suboptimal therapy, which likely explains the difference in duration of hospitalizations between both groups. Whereas patients infected with MSMp were hospitalized for a median of 2 days, those infected with MRMp had a median of 8 days of hospitalization. These data confirmed similar findings previously described in other countries (*16*,*27*,*29*) but differ from what was found by Waites et al. in a recent surveillance study, in which they did not find any differences in clinical severity between groups (*10*).

Previous studies have described longer duration of fever in patients infected with MRMp, as well as longer time to defervesce while receiving macrolide therapy, with subsequent quick defervescence when switched to effective therapy (*16*–*18*,*20*,*25*,*29*,*30*). In our pediatric cohort, we found no difference in duration of fever at the first medical encounter. In addition, we found that patients infected with MRMp had lower maximum temperatures, which differs from 2 studies from China that described higher fevers in pediatric patients infected with MRMp (*30*,*31*). Because of the retrospective design of our study, we were not able to compare the duration of fever while patients were receiving therapy because most hospitalized patients with MSMp were discharged while still febrile, and a large portion of our cohort was managed as outpatients. Despite this limitation, we documented that patients infected with MRMp were still symptomatic and seeking medical attention while already being treated with macrolides at higher rates when compared with patients infected with MSMp. Likewise, we observed that patients with MRMp infection were more likely to be treated with levofloxacin as an alternative/second-line therapy, which agrees with other reports (*10*,*17*,*18*,*29*). It is crucial to note that several studies mentioned that eventually these patients with MRMp became afebrile, even if they were still receiving macrolide therapy; however, their duration of illness and subsequent hospitalization were longer (*32*). Also, we documented that 196 (57%) patients in our cohort had >1 previous medical visit, and 111 (25.6%) received prescriptions for antimicrobial drugs during their visit. Most of those antimicrobial drugs (96%) did not target mycoplasma infections. None of those patients were tested for *M. pneumoniae* infection during this initial encounter, thus contributing to the lack of targeted therapy.

Emerging literature, mainly from Asia, reports that, in addition to longer duration of hospitalization, more severe disease and more complications have been observed in MRMp infected patients. Those studies described more common pleural effusions, worse lung infiltrates, extrapulmonary complications, increased oxygen requirement, and increased need for ICU admission (*16*,*33*,*34*). These findings, however, were not documented in our study, nor in the meta-analysis by Chen et al. (*20*). Even so, in our cohort we observed that patients with MRMp had 4- to 5-fold greater odds for PICU admission, after adjusting for other factors.

The use of a respiratory panel PCR in 176 (51%) patients in the cohort provided additional information for evaluating the interactions between *M. pneumoniae* and viral infections; previous studies on MRMp infections have not analyzed these interactions. The univariate analysis showed that among patients with MRMp infections, viral co-detection was more frequent in those who were not hospitalized. At this point we have no clear explanation for this finding.

Our study’s first limitations are that it was performed at a single center and was retrospective. The resistance rates we found were based only on patients who sought medical care, which could lead to a potential bias in our MRMp rate, because not all patients with *M. pneumoniae* infection may need medical attention. From the laboratory perspective, 54 (9.8%) samples that were culture positive were unable to be sequenced. We did not confirm all cultures by *M. pneumoniae* PCR, so it is possible that the culture result was falsely positive. In addition, culture positivity was determined by a color change in the culture medium. Therefore, the presence of any microbial growth could cause color change and be misinterpreted and falsely called *M. pneumoniae* positive, which could be the reason that they failed sequencing. Furthermore, because of the study design, in which we started with the *M. pneumoniae*–positive samples available in the laboratory, no clinical data were available in ≈50% of the samples analyzed. Despite these limitations, we included clinical information from 344 children infected with *M. pneumoniae*, which represents one of the largest contemporary pediatric cohorts published in the United States. Finally, we did not attempt to genotype our isolates of *M. pneumoniae*. Others have reported some association between emerging *p1* gene types and increased macrolide resistance (*35*).

Our study was not designed to address the indications for testing for *M. pneumoniae* in children with CAP, whether detection of *M. pneumoniae* in the upper respiratory tract indicates active infection, or whether antimicrobial drug therapy offers a clear benefit to all patients with *M. pneumoniae* infections. Despite the limitations of the retrospective design, our study showed that lack of specific testing for *M. pneumoniae* frequently led to empiric therapy with noneffective antimicrobial drugs and that children hospitalized with MRMp infections had a more prolonged clinical course until they were switched to appropriate therapy, suggesting that antimicrobial drug therapy did modify the course of the disease. Large, prospective, multicenter studies are needed to address these key questions to optimize the management of these frequent infections among the pediatric population.

In summary, the rate of MRMp infections in pediatric patients in central Ohio is low (2.8%). Despite this low rate, children hospitalized with MRMp infections had worse clinical outcomes, defined by longer duration of hospitalization and higher odds of PICU admission, than those with infected with MSMp. Although prevalence is low, clinicians should be aware of the possibility of MRMp infection, particularly in patients who do not show clinical improvement while on macrolide therapy.
